# Ovarian Tissue Removed with Endometrioma May Reflect the Quality of the Adjacent Ovary

**DOI:** 10.3390/healthcare13080948

**Published:** 2025-04-20

**Authors:** Francesco G. Martire, Veronica Yacoub, Eliana Fuggetta, Valerio Carletti, Lucia Lazzeri, Gabriele Centini, Claudia D’Abate, Giuseppe Sorrenti, Errico Zupi, Francesco Maneschi

**Affiliations:** 1 Department of Molecular and Developmental Medicine, Obstetrics and Gynecological Clinic, University of Siena, 53100 Siena, Italy; 2 Department of Obstetrics and Gynecology, Tor Vergata University, 00133 Rome, Italy; 3 Department of Obstetrics and Gynecology, San Giovanni Addolorata Hospital, 00184 Rome, Italy; 4 Department of Gynecology, San Carlo Nancy Hospital, 00165 Rome, Italy

**Keywords:** endometriosis, endometrioma, laparoscopic stripping, follicular score

## Abstract

**Background/Objectives**: Endometriosis is commonly associated with infertility due to multiple factors. In this paper, we investigated the histopathological factors underlying these effects by comparing microscopic samples obtained during laparoscopic stripping. **Methods**: Morpho-functional examination through the follicular Score (FS) of ovarian tissue adjacent to the cystic wall (Specimen 1) was compared with the FS of ovarian tissue inadvertently harvested during cystectomy (Specimen 2). The follicular score was compared with clinical factors such as age, parity, BMI, and CA-125 levels. **Results**: Cohen’s kappa analysis revealed a 77.8% concordance between the follicular score of the ovarian tissue alongside the endometrioma (S1) and the ovarian tissue inadvertently removed (S2), reflecting a moderate level of concordance between the two samples. A statistically significant positive correlation was observed between the FS of Specimen 1 and the preoperative CA-125 value (*p* = 0.01); in contrast, a negative correlation was found between the FS and both the patient’s age (*p* = 0.006) and parity (*p* = 0.03). Additionally, a statistically significant negative correlation was demonstrated between the FS of Specimen 2 and patient age (*p* = 0.04). **Conclusions**: The functional quality of the remaining ovary after endometrioma stripping may be assessed by evaluating the follicular score of the pericystic ovarian tissue.

## 1. Introduction

Endometriosis is a chronic condition with early onset, characterized by the presence of endometrial tissue outside the uterine cavity, which can cause inflammation, infertility, and is frequently associated with symptoms such as dyspareunia, dysmenorrhea, and chronic pelvic pain [1,2]. The prevalence of endometriosis among women of reproductive age is 10–15% [3]. The ovary is the most common site for endometriosis, with ovarian endometrioma (OMA) occurring in 17% to 44% of affected patients [4]. Currently, medical management for endometriosis-associated pelvic pain is considered the standard therapeutic approach [5]. Therefore, surgery remains indicated to reduce endometriosis-associated pain, particularly in women with large cysts as well as those who decline long-term medical therapy, experience significant adverse effects, or have contraindications to hormonal treatment [6].

Minimally invasive surgery is considered the gold standard for treating endometriosis. Laparoscopic excision using the stripping technique has been established as the gold standard in the surgical management of ovarian endometriomas [7,8,9,10]. However, it has been claimed that surgery may adversely affect the ovarian microhistological and microanatomical function due to the unintended coagulation or excision of healthy ovarian tissue alongside the endometrioma wall, resulting in a reduction in follicular reserve [11,12,13,14].

It is well established that endometriomas compromise the quality of ovarian tissue, leading to alterations in follicular and vascular patterns [15]. Indeed, the ovarian tissue excised with the endometrioma wall typically lacks the histological and potentially functional characteristics of normal ovarian tissue [16]. Previous studies suggest that the residual ovarian tissue may resemble the tissue adhering to the excised cyst; however, there is currently no conclusive evidence to support this hypothesis.

Therefore, this study aims to compare the histopathological and morphological characteristics of the follicular patterns in ovarian tissue removed from the cystic wall with those of ovarian tissue inadvertently harvested during cystectomy, considering the latter as a sample of the entire remaining ovary.

## 2. Materials and Methods

The study was performed in the Department of Obstetrics and Gynecology at S.M. Goretti Hospital in Latina and San Giovanni Addolorata Hospital in Rome, from January 2015 to December 2020. A retrospective analysis was performed on premenopausal patients who had a clinical and ultrasonographic diagnosis of ovarian endometrioma and underwent laparoscopic stripping procedure according to European Society of Human Reproduction and Embryology (ESHRE) guidelines at the time [17]. The inclusion criteria consisted of the following: women of reproductive age between 18 and 45 years, with a Body Mass Index (BMI) ranging from 18 to 30 kg/m^2^, presenting with unilateral or bilateral endometriomas exceeding 30 mm in diameter, as confirmed by transvaginal ultrasonography. None of the patients had received medical therapy for at least three months before surgery. All patients reported dysmenorrhea, dyspareunia, or chronic pelvic pain with a Numeric Rating Scale (NRS) score greater than seven, which constituted the main indication for surgical treatment. Exclusion criteria included the use of hormonal therapy within three months before surgery, any major chronic illness, previous ovarian or pelvic surgery, and a histologically unconfirmed diagnosis of endometrioma. Informed consent was obtained from all patients. Therefore, fifty-two patients were enrolled in the study. The study was conducted in accordance with the 2013 Declaration of Helsinki. All pelvic ultrasound examinations were performed by experienced gynecologists with a minimum of 15 years of experience in gynecological ultrasonography. Upon admission, detailed medical, gynecological, and obstetric histories were collected. Clinical data were extracted from patients’ records and paraffin blocks of pathological specimens were subsequently re-evaluated. The following parameters were documented: patient age, parity, BMI, CA-125 levels, and cyst diameter.

Laparoscopic excision of the ovarian cyst was performed as reported elsewhere, utilizing the stripping technique [18]. Briefly, following the induction of general anesthesia, a 10 mm laparoscope was introduced through the umbilicus, and three accessory 5 mm trocars were placed suprapubically to allow the introduction of ancillary instruments. Following an initial diagnostic assessment of the pelvis and abdomen, peritoneal washing was performed for cytological examination. During ovarian mobilization, upon rupture of the cystic wall, complete aspiration of the cystic contents was performed. Following meticulous identification of the cleavage plane, the cystic wall was separated from the remaining ovarian parenchyma using blunt and sharp dissection, while gentle traction was applied in opposing directions with atraumatic forceps to preserve the visible follicles on the surface of the remaining ovary, particularly near the hilum. Hemostasis was achieved using pinpoint bipolar coagulation to manage small bleeders. No sutures were applied to the ovarian parenchyma, and the cystic wall was retrieved from the abdominal cavity using an endobag. All cystectomies were performed by a single expert surgeon (FM). Patients were discharged on the 2nd postoperative day in the absence of adverse events. The cyst wall was sent for routine histological examination and designated as Specimen 1. In instances where the pathologist identified small fragments of ovarian tissue not associated with the cyst wall, these were also submitted for histological analysis and designated as Specimen 2, representing healthy ovarian tissue inadvertently harvested during the surgical procedure. Surgical specimens were fixed in 10% buffered formalin for no less than 2 h and no more than 20 h, followed by rinsing in running water and dehydration in increasing concentrations of ethyl alcohol. The specimens were then cleared in xylene, infiltrated, and embedded in paraffin. The dehydration, clearing, and infiltration processes were conducted under vacuum conditions. Consecutive sections of approximately 5 μm thickness were obtained using a microtome and stained with hematoxylin–eosin.

The diagnostic criteria established by the American College of Obstetricians and Gynecologists (ACOG) were followed to diagnose endometriosis. Specifically, the presence of two or more of the following histological features was assessed: endometrial glands, endometrial stroma, or hemosiderin-laden macrophages [19].

The follicular scores of both specimens were evaluated using a semi-quantitative scoring system as follows [14]: G0 = complete absence of follicles; G1 = presence of only primordial follicles; G2 = presence of primordial and primary follicles; G3 = presence of some secondary follicles; G4 = presence of primary and secondary follicles in a pattern consistent with normal ovary.

### Statistical Analysis

Descriptive statistics, including means and standard deviations for continuous variables and percentage changes for repeated measures, were employed to characterize the study sample. A *t*-test was utilized to compare continuous parametric variables, while the chi-squared test was applied for non-parametric variables. Additionally, Spearman’s rank correlation coefficient was used to assess the association between variables. Cohen’s kappa coefficient was employed to evaluate the concordance between the follicular scores of Specimen 1 and Specimen 2. A *p*-value of less than 0.05 was considered statistically significant. All analyses were performed using the Statistical Package for the Social Sciences (SPSS) version 22.0 for Mac (SPSS, Chicago, IL, USA).

## 3. Results

Ninety-eight consecutive patients aged 25–45 years were assessed for eligibility prior to surgery. Among all patients assessed, the endometriotic nature of cysts was confirmed histopathologically in 83 cases. A separate ovarian specimen, consisting of identifiable ovarian tissue (stroma with follicles) inadvertently harvested, was available in 52 (62%) of the 83 patients. In 52 women both Specimens 1 and Specimen 2 were available, and all 52 patients were enrolled in the study (Table 1).

The mean age was 34.31 ± 5.77 years (range, 25–45). The mean BMI was 22.33 ± 3.40 kg/m^2^. The mean preoperative CA-125 value was 67.38 ± 74.62 U/mL. The mean cyst size for all treated cysts was 45.1 ± 20.3 mm, with a maximum cyst wall thickness of 1.2 ± 0.4 mm. Demographic and clinical data for the patients are summarized in Table 1. The prevalence of different FS for both groups is reported in Table 2. In Group 1, we observed 14 patients with G0 follicles, 16 with G1 follicles, 14 with G2 follicles, 8 with G3 follicles, and 0 with G4 follicles. In Group 2, we observed 18 patients with G0 follicles, 16 with G1 follicles, 8 with G2 follicles, 10 with G3 follicles, and 0 with G4 follicles (Table 2).

From both a functional (G0 and G1 represent follicles with poor functional significance for fertility purposes) and a statistical (considering the small sample size) perspective, we grouped G0–G1 follicles as having poor oocyte quality and G2, G3, G4 follicles as having good oocyte quality. No statistically significant relationship was observed between the follicular score (FS) of pericystic ovarian tissue (Specimen 1) and the patient’s BMI (*p* = 0.37) or cyst size (*p* = 0.2). Conversely, a significant positive correlation was found between the FS of Specimen 1 and the preoperative CA-125 value (*β* = 0.14, *p* = 0.01). Additionally, a negative correlation was observed between the FS of Specimen 1 and both patient age (*β* = −0.36; *p* = 0.006) and parity (*β* = 0.32, *p* = 0.03) (Table 3).

Furthermore, no statistically significant relationship was observed between the follicular score of healthy ovarian tissue inadvertently excised during surgery (Specimen 2) and the patient’s BMI (*p* = 0.14), parity (*p* = 0.32), preoperative CA-125 value (*p* = 0.25), or cyst size (*p* = 0.11). However, a statistically significant relationship was found between the FS of Specimen 2 and patient’s age (*β* = −0.21; *p* = 0.04) (Table 4).

Lastly, Cohen’s concordance analysis indicated a 77.8% agreement between the follicular scores of Specimens 1 and Specimen 2, with a kappa coefficient of 0.56, reflecting moderate concordance between the two specimens.

## 4. Discussion

Endometriosis is a chronic disease with a significant impact on quality of life. While medical therapy provides clear benefits for pain management, as far as fertility is concerned, we do not know how endometrioma has a direct effect on healthy ovarian parenchyma. Although it is widely acknowledged in the literature that endometriosis is associated with infertility through several mechanisms [20], no direct evidence has been provided regarding the follicular score of the remaining ovary. Previous studies have not analyzed ovarian tissue inadvertently removed during surgery. Several factors may be involved in this process, including cyst size, how long the cyst has been present, and patient age.

Therefore, the aim of this study was to try to understand how some characteristics of the endometrioma can impact the adjacent ovarian parenchyma and suggest adequate management to reduce fertility impairment.

In our study of 98 patients with ovarian endometriosis who underwent excision via stripping technique, healthy ovarian parenchyma was found in 52 (53.1%). This finding is in agreement with the literature. In fact, it has been reported that during the excision of endometriotic cysts, the rate of healthy ovarian tissue inadvertently excised from adjacent cysts (56–100%) [21] and the thickness of this tissue (1.7–3.5 mm) is significantly greater compared to the excision of benign cysts, such as dermoid cysts, serous cystadenomas, and mucinous cystadenomas (6–82%; 2.5–1.5 mm) [14]. This phenomenon may be due to an enhanced pericystic inflammatory environment and the increased difficulty of identifying the appropriate cleavage plane [15,16]. Considering these data, it is important to highlight that surgical treatment damages healthy ovarian parenchyma in approximately half of cases, potentially impairing fertility. Furthermore, OMA surgery may cause local inflammation and vascular damage due to surgical coagulation [22]. Regarding the effect of surgery on ovarian reserve, the literature remains controversial. Some studies have reported a significant decrease in ovarian reserve markers after surgery [12,23,24]. In contrast, the meta-analysis by Muzii et al. found no significant changes in antral follicle count (AFC) after surgery for ovarian endometriomas [25]. These inconsistent findings may be explained by the destruction of healthy ovarian tissue, which is not easily monitored and warrants further investigation. Additionally, in women with OMA, the reduction in Anti-Müllerian Hormone (AMH) and AFC levels is also influenced by the surgical technique used [26] and the surgeon’s experience [22]. Some authors argue that a decrease in AMH levels is reversible after 12 months [26], while others claim it is irreversible [24]. Additionally, when examining the histology of OMA cystectomy in relation to ovarian reserve, some studies have found that the ovarian tissue removed along with the endometriomas was primarily fibrotic or non-functional [21]. Recent research suggests that CO₂ laser ablation leads to a significant increase in AFC in the treated ovary, in contrast to the results observed following cystectomy [27].

However, endometriosis also reduces ovarian reserve [28], and the management of ovarian endometriomas remains controversial when considering reproductive outcomes in affected women.

In fact, a consistent and significant reduction in AMH levels was also observed in women with endometriosis who did not undergo surgical treatment (β = −0.66 ± 0.28) [29]. This phenomenon has been linked to ovarian anatomical distortion and an inflammatory environment [15], which is known to involve inflammatory mediators such as Interleukins −1, −8, Tumor Necrosis Factor, Transforming Growth Factor, and Interferon-Gamma [30]. Other studies have found reduced follicle density in the ovarian cortex surrounding the endometrioma, indicating that the endometrioma itself may cause direct ovarian damage [31]. Furthermore, women undergoing IVF with OMA exhibit lower levels of reproductive variables—including AMH, follicle count, number of oocytes, and number of embryos—compared to women with infertility unrelated to OMA [23]. Moreover, reproductive and pregnancy outcomes were not significantly influenced by surgical treatment of OMA. These findings may have important implications for obstetric outcomes in young patients with endometriosis who undergo surgery. Another important aspect of our study was the concordance (78%) between the morpho-functional characteristics of the ovarian tissue surrounding the cystic wall and those of the remaining ovarian tissue.

According to these data, the status of the remaining ovary may be indirectly assessed by examining the ovarian tissue excised from the cyst, and its follicular score may reflect the functional status of the remaining ovary. These findings on the quality of the residual ovary provide valuable insights for subsequent fertility management. While current guidelines state that laparoscopic treatment can increase spontaneous pregnancy rate [4], using our data we may follow different paths. For instance, in patients with a poor FS, the gynecologist may consider immediate Assisted Reproductive Technology (ART), whereas in those with a good FS, the possibility of attempting spontaneous conception in the following months may be explored. Therefore, the analysis of ovarian tissue inadvertently excised during surgery may serve as a reference for assessing the functional status of the entire ovary. Our results suggest a potential approach for estimating overall ovarian function by analyzing only pericystic ovarian tissue. According to these data, we aimed to evaluate how certain cyst characteristics and patient factors influence the adjacent ovarian parenchyma. Several findings originate from the pericystic ovarian tissue (Specimen 1). The FS observed in both specimens demonstrated a significant negative correlation with patient age. In the literature, patient age is widely recognized as a key factor influencing the deterioration of ovarian reserve [32]. Given that endometriosis has an early onset, the presence of longstanding endometriotic lesions and increasing patient age may have a synergistic effect on oocyte quality deterioration [24]. Previous studies have shown a rapid decline in AMH levels and AFC in women with OMA compared to age- and BMI-matched women without endometriosis [33,34,35,36]. Our findings corroborate that age significantly impacts ovarian reserve quality, as assessed by the follicular score.

Conversely, no data are currently available regarding the correlation between patient parity and FS. The negative correlation observed in our study may reflect the advanced maternal age of parous patients in Italy (32.2 years, as reported by the Italian Health Ministry in 2020) resulting in a reduced FS. In the literature, it has been reported that the larger the cyst, the thicker the ovarian parenchyma removed along with the cystic wall due to the complexities of surgical procedures [37]. This tissue loss averages approximately 200 microns per centimeter increase in cyst diameter [14]. However, no studies have evaluated whether cyst diameter adversely affects the follicular quality of ovarian tissue. In this study, we did not find a significant association, but rather a trend. The small sample size may explain the lack of statistical significance.

Additionally, our study found a positive correlation between preoperative serum CA-125 levels and FS. CA-125 is a well-established marker of peritoneal inflammation synthesized by the coelomatic epithelium, which includes the endometrium, fallopian tubes, ovaries, and peritoneum [38]. In the context of endometriosis, CA-125 levels are up-regulated due to stimulation of the coelomatic epithelium, reflecting the state of peritoneal inflammation [39]. Our data suggest that high CA-125 levels may serve as a marker of ovarian health in patients with endometriosis. It has been proposed that both endometriosis and the early follicular phase are significant predictors of CA-125 levels. Specifically, blood samples drawn during the early follicular phase have been significantly associated with higher CA-125 levels, suggesting that fertile women may exhibit greater fluctuations in CA-125 levels. However, it is well known that CA-125 levels are nonspecific, as they are influenced by factors such as age, race, uterine fibroids, endometriosis, and ovarian neoplasms [40,41]. Thus, our data confirm, in accordance with the 2022 ESHRE guidelines [4], that CA 125 levels are highly nonspecific and more related to inflammatory status than to disease progression.

The limitations of the study include its monocentric design and the small number of specimens, which was constrained by ethical considerations.

Conversely, the strengths of this study lie in the fact that all procedures were performed by the same surgeon, ensuring surgical consistency, and that patients were specifically selected based on severe pelvic pain symptomatology.

## 5. Conclusions

Our findings suggest that the quality of ovarian tissue remaining after endometrioma stripping can be assessed by evaluating the follicular score of the pericystic ovarian tissue. This assessment may have significant reproductive implications, including its impact on conception rates and follicular recruitment during ovarian stimulation. Further studies are required to validate our data and confirm this hypothesis.

## Figures and Tables

**Table 1:** Demographic and clinical characteristics of 52 patients.

Parameter	Study Population (*n* = 52)
Age, year (mean ± SD)	34.3 ± 5.7
BMI, kg/m^2^ (mean ± SD)	22.3 ± 3.4
Parous ≥ 1 (number, %)	20 (38.4%)
Preoperative CA-125, UI/mL (mean ± SD)	67.3 ± 74.6
Cyst size, mm (mean ± SD)	45.1 ± 20.3

**Table 2:** Distribution of follicular score grading among the two specimens.

Cystic FS	Specimen 1, N (%)	Specimen 2, N (%)
G0	14 (27)	18 (35)
G1	16 (31)	16 (31)
G2	14 (27)	8 (15)
G3	8 (15)	10 (19)
G4	0 (0)	0 (0)

**Table 3:** Relationship between the follicular score of ovarian tissue found adjacent to the ovarian cyst (Specimen 1) and clinical variables (univariate analysis and Pearson correlation; dependent variable: cyst’s follicular ovarian score).

Variables	Cystic Follicular Score			
	0–1 (N. 30)	2–4 (N. 22)	*p*-Value	β-Coefficient
Age, year (mean ± SD)	36.1 ± 5.9	31.8 ± 4.2	0.006	−0.36
BMI, kg/m^2^ (mean ± SD)	21.9 ± 3.1	22.8 ± 3.5	0.37	/
Parous ≥ 1 (number, %)	20 (66.6%)	8 (36.3%)	0.03	−0.32
Preoperative CA-125 (mean ± SD)	45.2 ± 33.6	97.5 ± 98.9	0.01	0.14
Cyst size, mm (mean ± SD)	4.8 ± 2.4	4.1 ± 1.2	0.2	/

**Table 4:** Relationship between the follicular score of ovarian tissue inadvertently excised during surgery (Specimen 2) and clinical variables (univariate analysis and Pearson correlation; dependent variable: follicular score of healthy ovarian tissue).

Variables	Ovarian Tissue Follicular Score			
	0–1 (N. 34)	2–4 (N. 18)	*p*-Value	β-Coefficient
Age, year (mean ± SD)	35.4 ± 6.2	32.1 ± 3.6	0.04	−0.21
BMI, kg/m^2^ (mean ± SD)	21.8 ± 2.9	23.3 ± 3.8	0.14	/
Parous ≥ 1 (number, %)	20 (58.8%)	8 (55.5%)	0.32	/
Preoperative CA-125 (mean ± SD)	58.7 ± 68.6	83.7 ± 80.4	0.25	/
Cyst size, mm (mean ± SD)	4.8 ± 2.3	3.9 ± 1.0	0.11	/

## Data Availability

The data presented in this study are available on request from the corresponding author.

## Abbreviations

The following abbreviations are used in this manuscript:

BMI	Body Mass Index
FS	Follicular Score
OMA	Ovarian Endometrioma
US	Ultrasound
MRI	Magnetic Resonance Imaging
ESHRE	European Society of Human Reproduction and Embryology
ACOG	American College of Obstetricians and Gynecologists
AFC	Antral Follicle Count
AMH	Anti-Müllerian Hormone
NRS	Numeric Rating Scale
ART	Assisted Reproductive Technology
